# Multidisciplinary approach to the management of renal angiomyolipoma with inferior vena cava thrombus and pulmonary embolism: a case report

**DOI:** 10.1093/jscr/rjae174

**Published:** 2024-03-22

**Authors:** Jordyn Perdue, Alexandra Wells, Krishna Patel, Wihan Du Plessis, Jaya Varre, Patrick Salibi

**Affiliations:** Department of General Surgery, OhioHealth Riverside Methodist Hospital, Columbus, OH 43214, United States; Department of Hepato-Pancreato-Biliary (HPB) Surgery, Atrium Health, Charlotte, NC 28203, United States; Department of General Surgery, OhioHealth Riverside Methodist Hospital, Columbus, OH 43214, United States; Department of General Surgery, OhioHealth Riverside Methodist Hospital, Columbus, OH 43214, United States; Department of General Surgery, OhioHealth Riverside Methodist Hospital, Columbus, OH 43214, United States; Department of General Surgery, OhioHealth Riverside Methodist Hospital, Columbus, OH 43214, United States

**Keywords:** renal angiomyolipoma, renal thrombus, inferior vena cava thrombus, pulmonary embolism, multidisciplinary care

## Abstract

Renal angiomyolipoma (AML) is a benign tumor with rare venous extension. We present a case of a patient with renal AML with inferior vena cava (IVC) tumor thrombus and acute pulmonary embolism (PE). A 34-year-old female presented with chest pain. Imaging revealed a 5 cm right renal AML, with tumor thrombus into the renal vein and IVC, and acute left lower lobe PE. Right radical nephrectomy and caval thrombectomy were performed using intraoperative ultrasound. Rarely, these benign tumors generate thrombus with caval extension. The location of IVC thrombus guides surgical planning, which may involve suprahepatic IVC control or cardiopulmonary bypass. Early involvement of a multidisciplinary team with extensive preoperative planning can help achieve successful outcomes.

## Introduction

Renal angiomyolipoma (AML) is classically a benign tumor with low risk for extension and invasion. Renal AML is composed of fat, smooth muscle, and abnormal blood vessels, and is further divided into classic and epithelioid variants, of which epithelioid is accepted to be more aggressive [1]. These benign lesions rarely invade into adjacent venous structures, and are even more rarely associated with pulmonary embolism (PE). We present a case of a 34-year-old patient with renal AML with an associated inferior vena cava (IVC) tumor thrombus and acute PE.

## Case report

**Figure 1:**
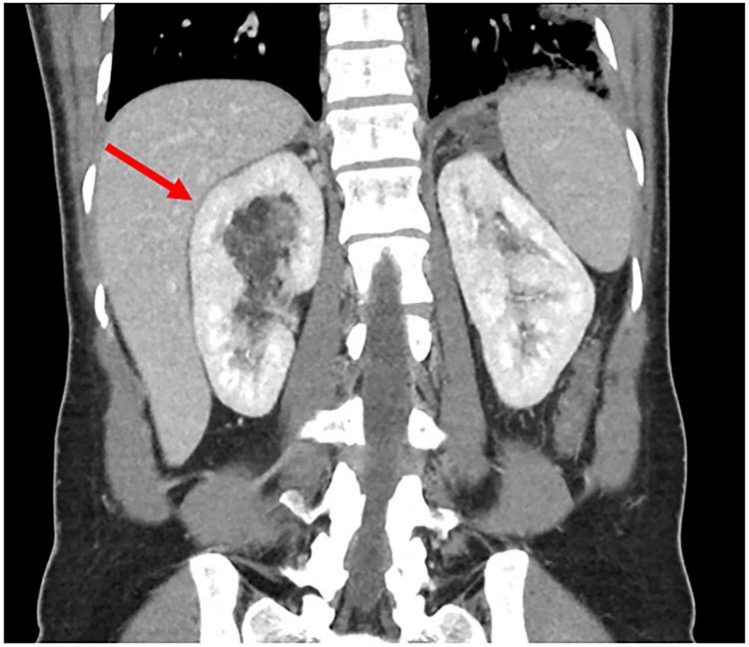
Preoperative CT showing renal AML.

**Figure 2:**
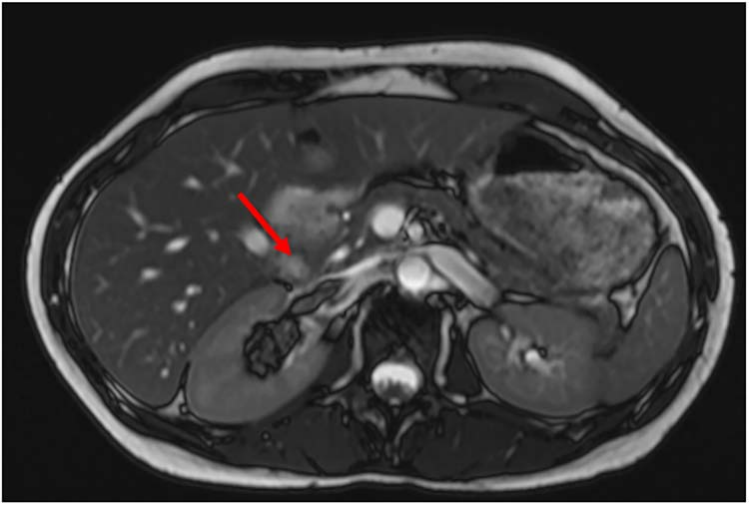
Preoperative MRI with tumor thrombus extension into right renal vein.

A 34-year-old female presented with acute onset left-sided chest pain and was found to have an occlusive PE in the left lower lobe. She was initiated on a heparin infusion. Imaging partially revealed a mixed-density lesion in the right kidney, which was further evaluated with a dedicated computed tomography (CT) scan and magnetic resonance imaging (MRI). This demonstrated concern for an AML involving the upper pole of the right kidney, with extension of tumor thrombus into the right renal vein and IVC (Figs 1 and 2). At this point, surgical oncology and cardiothoracic surgery (CTS) were involved to discuss resection. An echocardiogram confirmed that the tumor thrombus did not extend into the cavoatrial junction. MRI of the brain was ordered to rule out a rare presentation of tuberous sclerosis. Percutaneous renal biopsy demonstrated AML with epithelioid features. She was transitioned to therapeutic Lovenox and discharged with plans for surgical resection within the following week.

**Figure 3:**
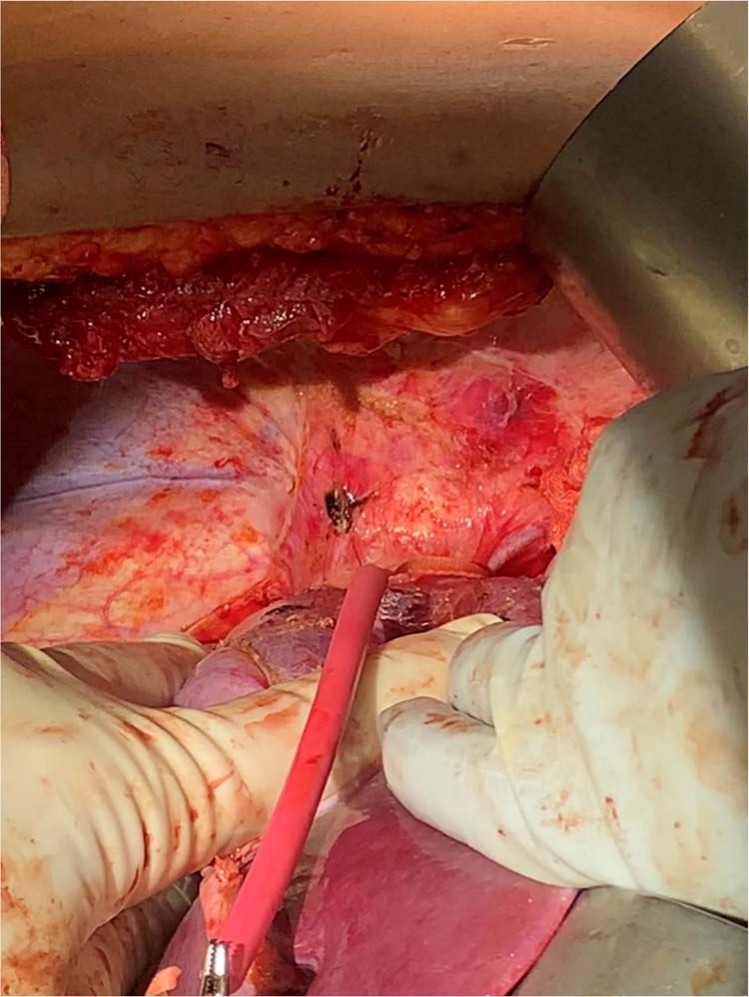
Suprahepatic IVC control.

Detailed interdisciplinary coordination took place between Surgical Oncology, CTS, and Cardiac Anesthesia teams preoperatively. CT angiogram was repeated, which did not show thrombus past the mid-hepatic IVC. Given these findings, it was determined the thrombus should be removable with the tumor in the abdomen. However, CTS was maintained on standby should the need for bypass arise. An exploratory laparotomy was performed via a subcostal incision. Intraoperative ultrasound confirmed tumor thrombus did not extend superior to the hepatic vein takeoff. The suprahepatic vena cava was controlled with a Rummel tourniquet (Fig. 3). Ultrasound was again used and confirmed that all thrombus was below the level of the tourniquet.

**Figure 4:**
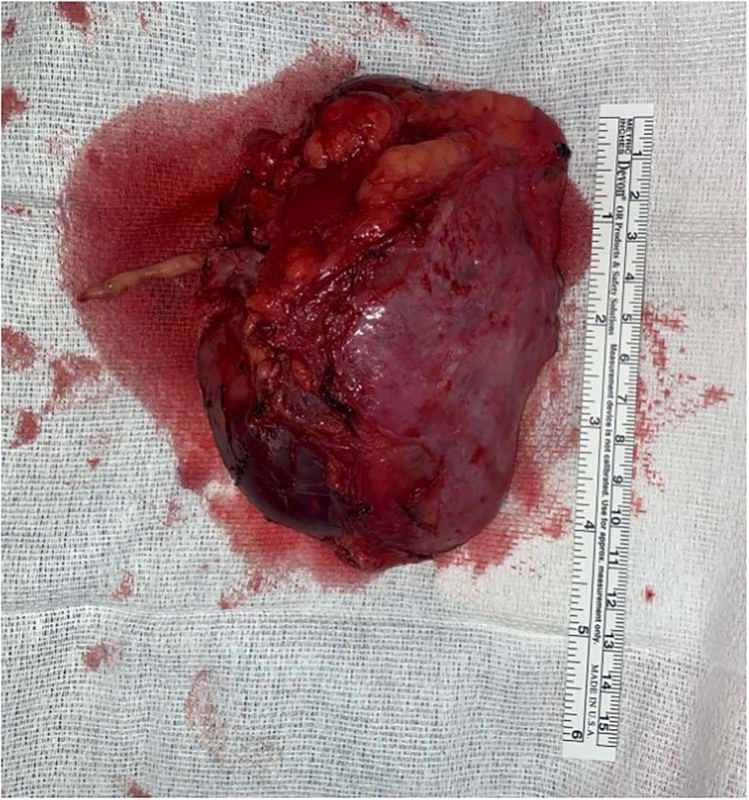
Specimen with thrombus extension into the right renal vein.

The left renal rein and infrarenal IVC were dissected and controlled. At this point, the right kidney was mobilized for en-bloc resection, sparing the right adrenal gland. The ureter was ligated, and the right renal artery transected with a vascular stapler. Intraoperative ultrasound and transesophageal echocardiogram (TEE) were used to ensure that mobilization had not shifted the tumor thrombus. CTS was then called into the OR for confirmation. After heparin circulation, the suprahepatic IVC, left renal vein, and infra-hepatic IVC were occluded. The right renal vein was excised from the IVC at its origin, and the thrombus milked out in retrograde fashion (Fig. 4). The caval opening was controlled with a Satinsky clamp, while infrarenal and suprahepatic IVC clamps released. The cava was widely patent and the venotomy was repaired primarily. Completion intraoperative ultrasound and TEE were performed, all demonstrating satisfactory flow without residual tumor thrombus. The patient had an unremarkable recovery and was discharged on postoperative day 6 with therapeutic Lovenox and eventual transition to oral anticoagulation.

## Discussion

Approximately 80% of renal AMLs present incidentally, with a minority of cases associated with genetic syndromes such as tuberous sclerosis or lymphangioleiomyomatosis [2]. The prevalence of renal AML varies from 0.2 to 0.6% of the population and is identified more frequently in women between the ages of 40 and 60 years old [2, 3]. Most lesions are identified incidentally by the classic imaging finding of fatty tumor with variable degrees of fat, blood vessels, and smooth muscle [2]. When symptoms are present, this is typically secondary to spontaneous hemorrhage, or more rarely flank pain, hematuria, or a palpable mass [1].

Renal AML with invasion of venous structures is a rare phenomenon, with <100 cases of IVC invasion reported [4]. Dissemination into PE is rarer, and only a few other cases have been identified [5–8]. Among renal cell carcinoma (RCC) tumors, involvement of renal vein or IVC ranges from 4 to 10%, with PEs being even less frequent [9]. Historically, renal AMLs >4 cm had been considered an indication for surgical removal given the increased risk for spontaneous hemorrhage, though this is being challenged in more recent literature [3].

Radical nephrectomy and thrombectomy remain the standard of care for renal tumors with tumor thrombus [9]. The location of IVC thrombus is important in both tumor staging and surgical planning, though different staging systems exist. One such classification system defines levels I–IV, with level I being <2 cm from renal vein, level II below the intrahepatic vena cava, level III within intrahepatic vena cava but below diaphragm, and level IV within atrium [9].

Traditionally, the management of suprahepatic or supradiaphragmatic vein thrombus includes cardiopulmonary bypass (CPB) or venovenous bypass [10, 11]. Bypass modalities mitigate the hypotension with suprahepatic IVC cross clamping, but can also lead to complications such as renal failure and endothelial dysfunction [12, 13]. Contrarily, non-bypass techniques can result in liver congestion, and inadequate clamping may shower tumor emboli [11, 13]. Prophylactic IVC filter placement is no longer recommended, though may be considered in cases with residual thrombus [14]. Mandhani et al. argues that the hepatic vein is a key anatomic landmark, as this necessitates clamping of the hepatoduodenal ligament to mitigate venous congestion [10]. At present, it appears most cases can be managed without CPB or VV bypass, unless there is atrial involvement [10, 12, 13]. Control of IVC via balloon occlusion is a new technique that shows promise in facilitating thrombus removal [15]. Given the variability of these patients’ perioperative courses, it is generally recommended that these surgeries be performed with specialized anesthesia teams, and in facilities with readily available CTS and vascular surgery [14].

This case further demonstrates the ability of a benign renal AML to generate tumor thrombus extending into IVC and causing a PE. More consideration should be given for these benign tumors to cause local invasion as well as systemic manifestations. Careful and collaborative planning involving surgical oncology, cardiothoracic, and vascular surgery teams is critical in optimizing patient outcomes.
